# *catena*-Poly[[tribenzyl­tin(IV)]-μ-4-formyl-2-meth­oxy-6-nitro­phenolato-κ^2^ *O* ^1^:*O* ^4^]

**DOI:** 10.1107/S1600536811016436

**Published:** 2011-05-07

**Authors:** Thy Chun Keng, Kong Mun Lo, Seik Weng Ng

**Affiliations:** a Department of Chemistry, University of Malaya, 50603 Kuala Lumpur, Malaysia

## Abstract

The formyl­meth­oxy­nitro­phenoxide ions in the polymeric title compound, [Sn(C_7_H_7_)_3_(C_8_H_6_NO_5_)]_*n*_, link adjacent triorganotin(IV) cations into linear chains lying close to (101) [Sn—O = 2.1227 (12) Å and Sn← O = 2.4936 (13) Å]. The Sn^IV^ atom is displaced out of the C_3_Sn girdle of the *trans*-C_3_SnO_2_ trigonal-bipyramidal polyhedron in the direction of the covalently-bonded O atom [Sn—O—C = 137.63 (11)°] by 0.247 (1) Å; the geometry is distorted towards an octa­hedron by a remote O atom of the meth­oxy subsituent [Sn⋯O = 3.019 (1) Å]

## Related literature

For a related structure, see: James *et al.* (1998 ▶). For a description of triorganotin phenoxides, see: Poller (1970 ▶).
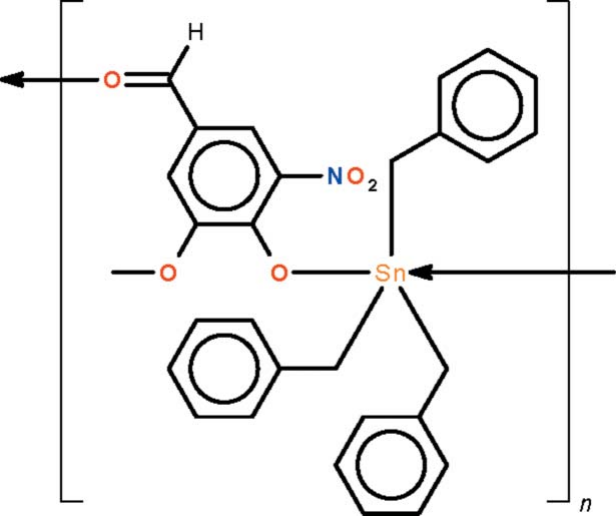


## Experimental

### 

#### Crystal data

[Sn(C_7_H_7_)_3_(C_8_H_6_NO_5_)]
*M* *_r_* = 588.21
Monoclinic, 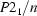

*a* = 12.1241 (1) Å
*b* = 16.5829 (2) Å
*c* = 13.3893 (2) Å
β = 106.3701 (6)°
*V* = 2582.83 (5) Å^3^
*Z* = 4
Mo *K*α radiation
μ = 1.03 mm^−1^
*T* = 100 K
0.25 × 0.25 × 0.10 mm

#### Data collection

Bruker SMART APEX diffractometer
Absorption correction: multi-scan (*SADABS*; Sheldrick, 1996 ▶) *T*_min_ = 0.783, *T*_max_ = 0.904
25760 measured reflections
6372 independent reflections
5545 reflections with *I* > 2σ(*I*)
*R*_int_ = 0.028

#### Refinement

*R*[*F* ^2^ > 2σ(*F* ^2^)] = 0.024
*wR*(*F* ^2^) = 0.063
*S* = 1.01
6372 reflections
325 parameters
H-atom parameters constrained
Δρ_max_ = 1.02 e Å^−3^
Δρ_min_ = −0.25 e Å^−3^

### 

Data collection: *APEX2* (Bruker, 2009 ▶); cell refinement: *SAINT* (Bruker, 2009 ▶); data reduction: *SAINT*; program(s) used to solve structure: *SHELXS97* (Sheldrick, 2008 ▶); program(s) used to refine structure: *SHELXL97* (Sheldrick, 2008 ▶); molecular graphics: *X-SEED* (Barbour, 2001 ▶); software used to prepare material for publication: *publCIF* (Westrip, 2010 ▶).

## Supplementary Material

Crystal structure: contains datablocks global, I. DOI: 10.1107/S1600536811016436/jh2283sup1.cif

Structure factors: contains datablocks I. DOI: 10.1107/S1600536811016436/jh2283Isup2.hkl

Additional supplementary materials:  crystallographic information; 3D view; checkCIF report

## Figures and Tables

**Fig. 1.**
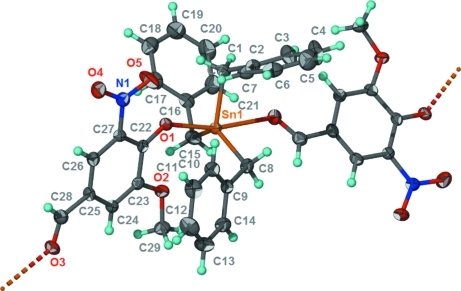
Thermal ellipsoid plot (Barbour, 2001) of a portion of the chain structure of Sn(C7H7)3(C8H6NO5) at the 70% probability level; hydrogen atoms are drawn as spheres of arbitrary radius.

## supplementary crystallographic information

### Comment

Triorganotin alkoxides are compounds, unlike triorganotin phenoxides, which are generally not stable in air, as stated in a textbook on organotin chemistry (Poller, 1970). As such, there are few reports on phenoxides. Vanillin and its derivatives possess a hydroxy group that can be deprotonated; the 5-nitrovallinate anion in polymeric [Sn(C_7_H_7_)_3_(C_8_H_7_NO_5_)]*_n_* (Scheme I) links adjacent triorganotin(IV) cations to form a linear chain running along the *a*–*c* diagonal of the monoclinic unit cell (Fig. 1). The dative Sn–O bond is significantly longer than the covalent Sn–O bond [Sn–O 2.123 (1), Sn←O 2.494 (1) Å], so that the Sn^IV^ atom is displaced out of the C_3_Sn girdle of the *trans*-C_3_SnO_2_ trigonal bipyramidal polyhedron in the direction of the covalently-bonded O atom [Sn–O–C 137.6 (1) Å] by 0.247 (1) Å. The geometry is distorted towards an octahedron by the O atom of the methoxy subsituent [Sn···O 3.019 (1) Å]. There are few examples of triorganotin systems having Sn←O_aromatic aldehdye_ bond; one example is triphenyltin salicylaldehydate, which features a long Sn←O bond (James *et al.*, 1998).

### Experimental

Tribenzyltin hydroxide was prepared by the base hydrolysis of tribenzyl)tin chloride with 10% sodium hydroxide solution. The compound (0.41 g, 1 mmol) hydroxide and 4-hydroxy-3-methoxy-5-nitrobenzaldehyde (5-nitrovanillin) (0.2 g, 1 mmol) were dissolved in ethanol (100 ml) and the mixture was heated for an hour. The solution was filtered and the solvent allowed to evaporate for a few days.

### Refinement

Carbon-bound H-atoms were placed in calculated positions (C—H 0.95 to 0.99 Å) and were included in the refinement in the riding model approximation, with *U*(H) set to 1.2 times *U*_eq_(C). The final difference Fourier map had a peak in the vicinity of Sn1.

### Crystal data

**Table:**

[Sn(C_7_H_7_)_3_(C_8_H_6_NO_5_)]	*F*(000) = 1192
*M**_r_* = 588.21	*D*_x_ = 1.513 Mg m^−^^3^
Monoclinic, *P*2_1_/*n*	Mo *K*α radiation, λ = 0.71073 Å
Hall symbol: -P 2yn	Cell parameters from 9964 reflections
*a* = 12.1241 (1) Å	θ = 2.4–28.2°
*b* = 16.5829 (2) Å	µ = 1.03 mm^−^^1^
*c* = 13.3893 (2) Å	*T* = 100 K
β = 106.3701 (6)°	Block, colorless
*V* = 2582.83 (5) Å^3^	0.25 × 0.25 × 0.10 mm
*Z* = 4	

### Data collection

**Table:**

Bruker SMART APEX diffractometer	6372 independent reflections
Radiation source: fine-focus sealed tube	5545 reflections with *I* > 2σ(*I*)
graphite	*R*_int_ = 0.028
ω scans	θ_max_ = 28.3°, θ_min_ = 2.0°
Absorption correction: multi-scan (*SADABS*; Sheldrick, 1996)	*h* = −16→16
*T*_min_ = 0.783, *T*_max_ = 0.904	*k* = −22→21
25760 measured reflections	*l* = −17→17

### Refinement

**Table:**

Refinement on *F*^2^	Primary atom site location: structure-invariant direct methods
Least-squares matrix: full	Secondary atom site location: difference Fourier map
*R*[*F*^2^ > 2σ(*F*^2^)] = 0.024	Hydrogen site location: inferred from neighbouring sites
*wR*(*F*^2^) = 0.063	H-atom parameters constrained
*S* = 1.01	*w* = 1/[σ^2^(*F*_o_^2^) + (0.0349*P*)^2^ + 0.888*P*] where *P* = (*F*_o_^2^ + 2*F*_c_^2^)/3
6372 reflections	(Δ/σ)_max_ = 0.001
325 parameters	Δρ_max_ = 1.02 e Å^−^^3^
0 restraints	Δρ_min_ = −0.25 e Å^−^^3^

### Fractional atomic coordinates and isotropic or equivalent isotropic displacement parameters (Å^2^)

**Table:**

	*x*	*y*	*z*	*U*_iso_*/*U*_eq_	
Sn1	0.368458 (10)	0.189545 (7)	0.722727 (9)	0.01643 (5)	
O1	0.27411 (11)	0.13103 (7)	0.81338 (10)	0.0204 (3)	
O2	0.28459 (11)	0.27891 (8)	0.88378 (11)	0.0218 (3)	
O3	−0.01389 (12)	0.27479 (8)	1.10347 (11)	0.0262 (3)	
O4	0.04948 (13)	−0.03179 (8)	0.87527 (11)	0.0296 (3)	
O5	0.15153 (15)	−0.00208 (10)	0.77303 (13)	0.0426 (4)	
N1	0.10833 (14)	0.01628 (9)	0.84212 (12)	0.0218 (3)	
C1	0.38886 (16)	0.07215 (11)	0.66076 (14)	0.0209 (4)	
H1A	0.4702	0.0645	0.6619	0.025*	
H1B	0.3691	0.0300	0.7052	0.025*	
C2	0.31382 (16)	0.06304 (11)	0.55183 (14)	0.0215 (4)	
C3	0.3568 (2)	0.07057 (14)	0.46631 (17)	0.0343 (5)	
H3	0.4368	0.0790	0.4768	0.041*	
C4	0.2842 (2)	0.06586 (17)	0.36588 (18)	0.0449 (6)	
H4	0.3150	0.0714	0.3084	0.054*	
C5	0.1676 (2)	0.05316 (15)	0.34835 (18)	0.0411 (6)	
H5	0.1183	0.0501	0.2793	0.049*	
C6	0.12380 (18)	0.04503 (12)	0.43205 (18)	0.0315 (5)	
H6	0.0438	0.0361	0.4208	0.038*	
C7	0.19544 (17)	0.04974 (11)	0.53232 (16)	0.0245 (4)	
H7	0.1639	0.0439	0.5893	0.029*	
C8	0.23785 (17)	0.26471 (12)	0.62210 (16)	0.0250 (4)	
H8A	0.2716	0.3188	0.6193	0.030*	
H8B	0.2191	0.2419	0.5510	0.030*	
C9	0.12731 (16)	0.27558 (12)	0.65014 (14)	0.0215 (4)	
C10	0.05093 (18)	0.21194 (12)	0.64538 (16)	0.0263 (4)	
H10	0.0688	0.1603	0.6236	0.032*	
C11	−0.05074 (17)	0.22273 (14)	0.67194 (17)	0.0305 (4)	
H11	−0.1013	0.1785	0.6689	0.037*	
C12	−0.07859 (19)	0.29776 (14)	0.70286 (18)	0.0350 (5)	
H12	−0.1483	0.3053	0.7209	0.042*	
C13	−0.00443 (18)	0.36165 (13)	0.70734 (18)	0.0325 (5)	
H13	−0.0233	0.4133	0.7285	0.039*	
C14	0.09776 (17)	0.35095 (12)	0.68101 (15)	0.0259 (4)	
H14	0.1479	0.3955	0.6841	0.031*	
C15	0.51642 (16)	0.22724 (12)	0.84500 (15)	0.0242 (4)	
H15A	0.5502	0.2766	0.8240	0.029*	
H15B	0.4943	0.2391	0.9092	0.029*	
C16	0.60161 (16)	0.15935 (12)	0.86363 (14)	0.0221 (4)	
C17	0.59103 (17)	0.09386 (12)	0.92531 (15)	0.0264 (4)	
H17	0.5317	0.0937	0.9589	0.032*	
C18	0.66561 (18)	0.02880 (13)	0.93860 (16)	0.0305 (4)	
H18	0.6574	−0.0152	0.9814	0.037*	
C19	0.75189 (19)	0.02786 (15)	0.88964 (17)	0.0346 (5)	
H19	0.8027	−0.0169	0.8981	0.042*	
C20	0.76353 (18)	0.09220 (15)	0.82865 (17)	0.0368 (5)	
H20	0.8227	0.0917	0.7949	0.044*	
C21	0.69000 (17)	0.15770 (14)	0.81585 (17)	0.0303 (4)	
H21	0.6999	0.2019	0.7741	0.036*	
C22	0.20411 (15)	0.15092 (11)	0.86575 (13)	0.0170 (3)	
C23	0.20697 (15)	0.23057 (11)	0.91021 (14)	0.0186 (3)	
C24	0.13811 (15)	0.25138 (11)	0.97130 (14)	0.0198 (4)	
H24	0.1428	0.3039	1.0007	0.024*	
C25	0.05980 (16)	0.19419 (11)	0.99044 (14)	0.0204 (4)	
C26	0.05257 (16)	0.11815 (11)	0.94786 (14)	0.0202 (4)	
H26	−0.0005	0.0802	0.9607	0.024*	
C27	0.12282 (15)	0.09688 (10)	0.88604 (14)	0.0180 (3)	
C28	−0.01506 (17)	0.21211 (12)	1.05483 (16)	0.0246 (4)	
H28	−0.0694	0.1721	1.0597	0.030*	
C29	0.28924 (18)	0.36164 (11)	0.91567 (16)	0.0258 (4)	
H29A	0.3482	0.3900	0.8920	0.039*	
H29B	0.3083	0.3645	0.9917	0.039*	
H29C	0.2143	0.3871	0.8851	0.039*	

### Atomic displacement parameters (Å^2^)

**Table:**

	*U*^11^	*U*^22^	*U*^33^	*U*^12^	*U*^13^	*U*^23^
Sn1	0.01783 (7)	0.01555 (7)	0.01806 (7)	0.00019 (4)	0.00854 (5)	0.00098 (4)
O1	0.0242 (7)	0.0177 (6)	0.0241 (7)	0.0014 (5)	0.0149 (5)	0.0016 (5)
O2	0.0257 (7)	0.0157 (6)	0.0285 (7)	−0.0025 (5)	0.0148 (6)	−0.0009 (5)
O3	0.0320 (8)	0.0237 (7)	0.0289 (7)	0.0005 (6)	0.0183 (6)	−0.0035 (6)
O4	0.0402 (8)	0.0228 (7)	0.0312 (8)	−0.0108 (6)	0.0186 (6)	−0.0012 (6)
O5	0.0593 (11)	0.0319 (9)	0.0528 (11)	−0.0168 (7)	0.0424 (9)	−0.0188 (7)
N1	0.0243 (8)	0.0199 (8)	0.0224 (8)	−0.0030 (6)	0.0088 (6)	−0.0005 (6)
C1	0.0230 (9)	0.0198 (9)	0.0219 (9)	0.0019 (7)	0.0098 (7)	−0.0002 (7)
C2	0.0256 (9)	0.0164 (8)	0.0234 (9)	0.0004 (7)	0.0087 (7)	−0.0003 (7)
C3	0.0347 (12)	0.0426 (13)	0.0299 (11)	−0.0121 (10)	0.0160 (9)	−0.0065 (10)
C4	0.0574 (16)	0.0559 (16)	0.0255 (11)	−0.0199 (13)	0.0183 (11)	−0.0060 (11)
C5	0.0479 (14)	0.0404 (13)	0.0281 (11)	−0.0067 (11)	−0.0004 (10)	−0.0039 (10)
C6	0.0280 (11)	0.0240 (10)	0.0402 (12)	0.0007 (8)	0.0056 (9)	−0.0034 (9)
C7	0.0276 (10)	0.0176 (9)	0.0309 (10)	0.0012 (7)	0.0125 (8)	−0.0024 (8)
C8	0.0273 (10)	0.0257 (10)	0.0260 (10)	0.0064 (8)	0.0138 (8)	0.0070 (8)
C9	0.0225 (9)	0.0246 (9)	0.0184 (9)	0.0054 (7)	0.0076 (7)	0.0040 (7)
C10	0.0304 (11)	0.0248 (10)	0.0231 (10)	0.0025 (8)	0.0066 (8)	−0.0007 (8)
C11	0.0239 (10)	0.0359 (11)	0.0307 (11)	−0.0049 (9)	0.0060 (8)	−0.0004 (9)
C12	0.0213 (10)	0.0457 (13)	0.0403 (13)	0.0029 (9)	0.0125 (9)	−0.0038 (10)
C13	0.0292 (11)	0.0290 (11)	0.0409 (12)	0.0078 (9)	0.0125 (9)	−0.0032 (9)
C14	0.0258 (10)	0.0240 (10)	0.0284 (10)	0.0040 (8)	0.0086 (8)	0.0021 (8)
C15	0.0236 (10)	0.0229 (9)	0.0271 (10)	−0.0038 (8)	0.0089 (8)	−0.0062 (8)
C16	0.0178 (9)	0.0262 (9)	0.0211 (9)	−0.0029 (7)	0.0036 (7)	−0.0053 (8)
C17	0.0224 (10)	0.0328 (11)	0.0234 (10)	−0.0047 (8)	0.0059 (7)	−0.0014 (8)
C18	0.0272 (10)	0.0323 (11)	0.0279 (10)	−0.0001 (8)	0.0009 (8)	0.0028 (9)
C19	0.0285 (11)	0.0413 (13)	0.0297 (11)	0.0103 (9)	0.0013 (9)	−0.0015 (10)
C20	0.0227 (10)	0.0572 (15)	0.0321 (11)	0.0088 (10)	0.0106 (9)	0.0027 (11)
C21	0.0227 (10)	0.0398 (12)	0.0299 (11)	−0.0001 (9)	0.0096 (8)	0.0061 (9)
C22	0.0193 (9)	0.0181 (9)	0.0143 (8)	0.0024 (7)	0.0056 (6)	0.0027 (7)
C23	0.0197 (9)	0.0172 (8)	0.0197 (9)	0.0003 (7)	0.0071 (7)	0.0035 (7)
C24	0.0244 (9)	0.0172 (8)	0.0191 (9)	0.0011 (7)	0.0084 (7)	0.0003 (7)
C25	0.0227 (9)	0.0221 (9)	0.0194 (9)	0.0015 (7)	0.0110 (7)	0.0015 (7)
C26	0.0209 (9)	0.0206 (9)	0.0220 (9)	−0.0010 (7)	0.0106 (7)	0.0025 (7)
C27	0.0213 (9)	0.0155 (8)	0.0182 (8)	0.0000 (7)	0.0072 (7)	−0.0004 (7)
C28	0.0274 (10)	0.0223 (9)	0.0291 (10)	0.0002 (8)	0.0159 (8)	0.0017 (8)
C29	0.0318 (11)	0.0165 (9)	0.0313 (10)	−0.0043 (8)	0.0125 (8)	−0.0019 (8)

### Geometric parameters (Å, °)

**Table:**

Sn1—O1	2.1227 (12)	C11—C12	1.383 (3)
Sn1—C15	2.1526 (19)	C11—H11	0.9500
Sn1—C1	2.1576 (18)	C12—C13	1.380 (3)
Sn1—C8	2.1619 (19)	C12—H12	0.9500
Sn1—O3^i^	2.4936 (13)	C13—C14	1.392 (3)
O1—C22	1.287 (2)	C13—H13	0.9500
O2—C23	1.357 (2)	C14—H14	0.9500
O2—C29	1.433 (2)	C15—C16	1.500 (3)
O3—C28	1.225 (2)	C15—H15A	0.9900
O3—Sn1^ii^	2.4936 (13)	C15—H15B	0.9900
O4—N1	1.233 (2)	C16—C17	1.392 (3)
O5—N1	1.223 (2)	C16—C21	1.395 (3)
N1—C27	1.451 (2)	C17—C18	1.387 (3)
C1—C2	1.493 (3)	C17—H17	0.9500
C1—H1A	0.9900	C18—C19	1.382 (3)
C1—H1B	0.9900	C18—H18	0.9500
C2—C3	1.391 (3)	C19—C20	1.375 (3)
C2—C7	1.402 (3)	C19—H19	0.9500
C3—C4	1.386 (3)	C20—C21	1.385 (3)
C3—H3	0.9500	C20—H20	0.9500
C4—C5	1.382 (4)	C21—H21	0.9500
C4—H4	0.9500	C22—C27	1.414 (2)
C5—C6	1.375 (3)	C22—C23	1.445 (3)
C5—H5	0.9500	C23—C24	1.367 (2)
C6—C7	1.380 (3)	C24—C25	1.416 (2)
C6—H6	0.9500	C24—H24	0.9500
C7—H7	0.9500	C25—C26	1.377 (3)
C8—C9	1.501 (3)	C25—C28	1.448 (3)
C8—H8A	0.9900	C26—C27	1.390 (2)
C8—H8B	0.9900	C26—H26	0.9500
C9—C10	1.394 (3)	C28—H28	0.9500
C9—C14	1.395 (3)	C29—H29A	0.9800
C10—C11	1.387 (3)	C29—H29B	0.9800
C10—H10	0.9500	C29—H29C	0.9800
			
O1—Sn1—C15	99.54 (6)	C12—C13—C14	120.5 (2)
O1—Sn1—C1	86.91 (6)	C12—C13—H13	119.8
C15—Sn1—C1	113.15 (7)	C14—C13—H13	119.8
O1—Sn1—C8	101.64 (6)	C13—C14—C9	120.71 (19)
C15—Sn1—C8	127.85 (8)	C13—C14—H14	119.6
C1—Sn1—C8	115.07 (8)	C9—C14—H14	119.6
O1—Sn1—O3^i^	166.47 (5)	C16—C15—Sn1	107.19 (12)
C15—Sn1—O3^i^	85.05 (6)	C16—C15—H15A	110.3
C1—Sn1—O3^i^	79.60 (6)	Sn1—C15—H15A	110.3
C8—Sn1—O3^i^	85.12 (6)	C16—C15—H15B	110.3
C22—O1—Sn1	137.63 (11)	Sn1—C15—H15B	110.3
C23—O2—C29	117.37 (14)	H15A—C15—H15B	108.5
C28—O3—Sn1^ii^	128.23 (13)	C17—C16—C21	117.91 (19)
O5—N1—O4	121.74 (16)	C17—C16—C15	120.65 (17)
O5—N1—C27	120.47 (15)	C21—C16—C15	121.36 (18)
O4—N1—C27	117.77 (15)	C16—C17—C18	121.16 (19)
C2—C1—Sn1	111.26 (12)	C16—C17—H17	119.4
C2—C1—H1A	109.4	C18—C17—H17	119.4
Sn1—C1—H1A	109.4	C19—C18—C17	120.1 (2)
C2—C1—H1B	109.4	C19—C18—H18	120.0
Sn1—C1—H1B	109.4	C17—C18—H18	120.0
H1A—C1—H1B	108.0	C20—C19—C18	119.4 (2)
C3—C2—C7	117.52 (18)	C20—C19—H19	120.3
C3—C2—C1	121.91 (17)	C18—C19—H19	120.3
C7—C2—C1	120.52 (17)	C19—C20—C21	120.8 (2)
C4—C3—C2	120.7 (2)	C19—C20—H20	119.6
C4—C3—H3	119.6	C21—C20—H20	119.6
C2—C3—H3	119.6	C20—C21—C16	120.6 (2)
C5—C4—C3	120.8 (2)	C20—C21—H21	119.7
C5—C4—H4	119.6	C16—C21—H21	119.7
C3—C4—H4	119.6	O1—C22—C27	123.00 (16)
C6—C5—C4	119.2 (2)	O1—C22—C23	121.05 (16)
C6—C5—H5	120.4	C27—C22—C23	115.93 (15)
C4—C5—H5	120.4	O2—C23—C24	126.31 (17)
C5—C6—C7	120.4 (2)	O2—C23—C22	111.73 (15)
C5—C6—H6	119.8	C24—C23—C22	121.95 (16)
C7—C6—H6	119.8	C23—C24—C25	119.54 (17)
C6—C7—C2	121.38 (19)	C23—C24—H24	120.2
C6—C7—H7	119.3	C25—C24—H24	120.2
C2—C7—H7	119.3	C26—C25—C24	120.43 (17)
C9—C8—Sn1	117.52 (13)	C26—C25—C28	117.20 (17)
C9—C8—H8A	107.9	C24—C25—C28	122.37 (17)
Sn1—C8—H8A	107.9	C25—C26—C27	120.06 (17)
C9—C8—H8B	107.9	C25—C26—H26	120.0
Sn1—C8—H8B	107.9	C27—C26—H26	120.0
H8A—C8—H8B	107.2	C26—C27—C22	122.06 (16)
C10—C9—C14	118.00 (18)	C26—C27—N1	116.78 (16)
C10—C9—C8	121.76 (18)	C22—C27—N1	121.16 (15)
C14—C9—C8	120.25 (18)	O3—C28—C25	125.14 (18)
C11—C10—C9	121.16 (19)	O3—C28—H28	117.4
C11—C10—H10	119.4	C25—C28—H28	117.4
C9—C10—H10	119.4	O2—C29—H29A	109.5
C12—C11—C10	120.2 (2)	O2—C29—H29B	109.5
C12—C11—H11	119.9	H29A—C29—H29B	109.5
C10—C11—H11	119.9	O2—C29—H29C	109.5
C13—C12—C11	119.5 (2)	H29A—C29—H29C	109.5
C13—C12—H12	120.2	H29B—C29—H29C	109.5
C11—C12—H12	120.2		
			
C15—Sn1—O1—C22	83.51 (18)	Sn1—C15—C16—C21	95.27 (19)
C1—Sn1—O1—C22	−163.52 (18)	C21—C16—C17—C18	−0.3 (3)
C8—Sn1—O1—C22	−48.54 (18)	C15—C16—C17—C18	176.55 (18)
O3^i^—Sn1—O1—C22	−167.57 (18)	C16—C17—C18—C19	−0.5 (3)
O1—Sn1—C1—C2	105.81 (13)	C17—C18—C19—C20	0.6 (3)
C15—Sn1—C1—C2	−155.13 (12)	C18—C19—C20—C21	0.0 (3)
C8—Sn1—C1—C2	4.40 (15)	C19—C20—C21—C16	−0.8 (3)
O3^i^—Sn1—C1—C2	−75.15 (13)	C17—C16—C21—C20	0.9 (3)
Sn1—C1—C2—C3	101.24 (19)	C15—C16—C21—C20	−175.92 (19)
Sn1—C1—C2—C7	−76.10 (19)	Sn1—O1—C22—C27	155.51 (14)
C7—C2—C3—C4	0.7 (3)	Sn1—O1—C22—C23	−26.3 (3)
C1—C2—C3—C4	−176.7 (2)	C29—O2—C23—C24	−5.3 (3)
C2—C3—C4—C5	−0.3 (4)	C29—O2—C23—C22	174.93 (15)
C3—C4—C5—C6	−0.1 (4)	O1—C22—C23—O2	3.8 (2)
C4—C5—C6—C7	0.2 (4)	C27—C22—C23—O2	−177.92 (15)
C5—C6—C7—C2	0.2 (3)	O1—C22—C23—C24	−176.01 (16)
C3—C2—C7—C6	−0.6 (3)	C27—C22—C23—C24	2.3 (3)
C1—C2—C7—C6	176.85 (17)	O2—C23—C24—C25	178.99 (17)
O1—Sn1—C8—C9	13.55 (16)	C22—C23—C24—C25	−1.2 (3)
C15—Sn1—C8—C9	−98.44 (16)	C23—C24—C25—C26	−0.1 (3)
C1—Sn1—C8—C9	105.59 (16)	C23—C24—C25—C28	179.63 (18)
O3^i^—Sn1—C8—C9	−178.30 (16)	C24—C25—C26—C27	0.3 (3)
Sn1—C8—C9—C10	−67.3 (2)	C28—C25—C26—C27	−179.43 (17)
Sn1—C8—C9—C14	113.34 (18)	C25—C26—C27—C22	0.8 (3)
C14—C9—C10—C11	−0.9 (3)	C25—C26—C27—N1	−178.67 (16)
C8—C9—C10—C11	179.72 (19)	O1—C22—C27—C26	176.18 (17)
C9—C10—C11—C12	0.6 (3)	C23—C22—C27—C26	−2.1 (3)
C10—C11—C12—C13	−0.2 (3)	O1—C22—C27—N1	−4.3 (3)
C11—C12—C13—C14	0.0 (3)	C23—C22—C27—N1	177.42 (15)
C12—C13—C14—C9	−0.3 (3)	O5—N1—C27—C26	167.06 (18)
C10—C9—C14—C13	0.7 (3)	O4—N1—C27—C26	−11.5 (2)
C8—C9—C14—C13	−179.89 (19)	O5—N1—C27—C22	−12.4 (3)
O1—Sn1—C15—C16	88.15 (13)	O4—N1—C27—C22	169.03 (17)
C1—Sn1—C15—C16	−2.56 (15)	Sn1^ii^—O3—C28—C25	−177.02 (14)
C8—Sn1—C15—C16	−158.91 (12)	C26—C25—C28—O3	175.07 (19)
O3^i^—Sn1—C15—C16	−79.02 (13)	C24—C25—C28—O3	−4.7 (3)
Sn1—C15—C16—C17	−81.43 (19)		

Symmetry codes: (i) *x*+1/2, −*y*+1/2, *z*−1/2; (ii) *x*−1/2, −*y*+1/2, *z*+1/2.
